# Evolution of breast cancer incidence in young women in a French registry from 1990 to 2018: Towards a change in screening strategy?

**DOI:** 10.1186/s13058-022-01581-5

**Published:** 2022-12-05

**Authors:** Yanis Hassaine, Emmanuelle Jacquet, Arnaud Seigneurin, Patricia Delafosse

**Affiliations:** 1 Cancer and Blood Diseases, Grenoble-Alpes University Hospital, Grenoble, France; 2 Registre du Cancer de L’Isère, Grenoble, BP 217, 38043 Grenoble Cedex 09, France; 3 Pôle Santé Publique, Service d’évaluation Médicale, Centre Hospitalier Universitaire Grenoble Alpes, BP 217, 38043 Grenoble Cedex 09, France

**Keywords:** Breast cancer, Young women, Risk factors, Incidence, Survival, Subtypes

## Abstract

**Background:**

The worldwide incidence of invasive breast cancer in women is increasing according to several studies. This increase in incidence seems to be higher in young women (< 40 years). However, the reasons for this trend are poorly understood. This article aims to provide the most recent estimates of this trend and assess whether there is indeed an increase in the incidence of breast cancer among young women to strengthen prevention campaigns.

**Methods:**

We collected data from the Isere cancer registry in France of all invasive breast cancers from January 1990 to December 2018. The standardized incidence rate was calculated for four age groups (< 40 years, 40–49 years, 50–74 years, ≥ 75 years) for this period. The 10-year relative survival was evaluated for each age group age for two periods (1990–1999 and 2000–2008). From 2011 to 2013, we analyzed the incidence and 5-year relative survival by tumor subtype (triple negative, luminal, HER2 amplified) for each age group.

**Results:**

A total of 23,703 cases were selected, including 1343 young women (< 40 years). The incidence of invasive breast cancer increased annually by 0,8% (95% CI 0,7; 1) in all age groups combined from 1990 to 2018. The highest incidence increase is found among young women, by 2,1% annually (95% CI 1,3; 2,8). Regarding tumor subtypes from 2011 to 2018, the incidence of triple negatives increases higher in young women (+ 1,4% by year, 95% CI − 8,2; 11) and those over 75 years (+ 4% by year, 95% CI − 5,1; 13,2), but the results are not statistically significant. 10-year relative survival in young women increased from 74,6% (95% CI 69,6; 78,9) to 78,3%(95% CI 73,7; 82,1) between 1990–1999 and 2000–2008, respectively. Five-year relative survival is better in young women among triple negative and HER2 amplified.

**Conclusion:**

Our study confirms the current trend of increasing the incidence of breast cancer in young women, associated with improved survival very likely attributable to earlier diagnosis due to increased awareness, and improvements in treatment. A better individualized risk-based screening strategy is needed for these patients. Additional studies will be needed to more accurately assess the risk of developing breast cancer and improve diagnostic performance.

## Introduction

Breast cancer (BC) is the most common tumor in women worldwide. It amounted to 25% of all women’s cancers globally and constitutes a real public health problem. The age-standardized incidence of BC is highly variable, ranging from < 40/100,000 person-years based on the world standard population (Central and East Asia and Africa) to > 80/100,000 (Australia, North America, and Western Europe). Although its incidence is more important in many developed countries, mortality rates are higher in countries with a low level of development [1].

The incidence of all cancers is increasing worldwide regardless of age [1]. Concerning BC, the incidence rate is increasing in several Western countries (the USA, Europe, Australia, etc.) for the last three decades [2, 3].

In France, it represents 33% of women’s cancer with around 59,000 new cases in 2018 [4]. It is the leading cause of cancer death in women closely followed by lung cancer [5]. BC screening programs have been implemented throughout the country since 2004 but started gradually since other 1994. It is recommended only in women aged 50 to 74 years or if there are risk factors such as a family history or a genetic mutation such as BRCA [6]. After an increase in incidence rates between 1990 and 2003, there was a stabilization until 2010 probably due to a diminution in menopausal hormonal prescriptions after 2003 and the screening saturation effect [7, 8]. Since this year, the incidence rate is rising (0,6% per year).

Mortality rates have been constantly decreasing over the last three decades (1,3% per year) thanks to therapeutic advances with the earlier diagnostic due to screening [5].

Among young women (YW), defined by the European consensus treatment guidelines as women aged 40 years or below, there is a significant increase in incidence rates of BC at least since the 1990s in Europe [9, 10]. This age group represents 5% of diagnoses in France and 7% worldwide [11]. Nevertheless, except for individuals with a high genetic risk, BC occurring in younger women remains poorly understood. However, recent studies show that lack of physical activity, alcohol, tobacco, age > 30 years of pregnancy, or a history of chest irradiation are several risk factors [12, 13].

The usual presentation of breast cancer in young women (BCYW) is later stages at diagnosis, more aggressive pathological characteristics, a higher rate of triple-negative and HER2-overexpressing tumors, and greater rates of recurrence in comparison with older women [14].

In France, from 1990 to 2018 incidence rate increased more in YW in comparison with all age groups combined (0,9% vs. 0,6% by year, respectively), but there are no data by histological subtype [5].

The increase in incidence of BCYW is worrying because the behavior of these tumors is in the majority of cases more aggressive in comparison with older women [15].

This study aimed to assess the evolution of incidence and the survival rates by tumor subtypes of BC among YW from the year 1990 to 2018 according to data from the registry of the department of Isere in France to improve awareness and prevention campaigns.

## Materials and methods

### Study design

This retrospective observational study was based on data from an ongoing population-based cancer registry in Isère, a French administrative entity with nearly 1.2 million inhabitants. It is a department located in the southeast of France, in a mountainous region where the city of Grenoble is the prefecture.

### Study population

We included women with invasive BC in the Isère department from January 1990 to December 2018.

All first incident female BC was included. Breast sarcomas, lymphomas, and carcinomas in situ were excluded.

### Data collection

Data were collected by the Isère Cancer Registry, which collects incident cancer cases from different sources including histopathology laboratories, oncology departments, social security offices, and medical databases.

The following variables were used: dates of birth and diagnosis as well as cancer site and tumor morphology according to the International Classification of Disease for Oncology, ICD-O.

From 2011, supplementary clinical data were collected for each case: the hormone receptor (estrogen receptor (ER), progesterone receptor (PR)) and the human epidermal growth factor receptor type 2 (HER2) status of the tumor. Therefore, we defined four groups according to the tumor subtype: luminal (RH +), triple negative (RH- and Her2-), Her 2 amplified (Her2 + , RH-), and patients with no information on tumor subtype.

For cases diagnosed between 1990 and 2010, information on tumor subtypes was not available in the registry database.

### Statistical analyses

Annual world standardized incidence rates were calculated for each calendar year from 1990 to 2018. We then computed incidence rates by calendar years among four age groups: < 40 years (young women), 40–49 years, 50–74 years (women invited to organized screening), and 75 years and over. Annual world standardized incidence rates were also computed by tumor subtypes from 2011 to 2018.

Incidence rates for all BC during the 1990–2018 period were modeled using Poisson regression with restricted cubic splines to model age, period, and cohort effects. The number of degrees of freedom for the cubic splines was determined considering the AIC criterion. The average annual percent change (AAPC) of incidence rates was computed using the model coefficients, and its 95% confidence intervals were obtained with the delta method. Separate Poisson regressions were used to model incidence rates for luminal, Her2, and triple-negative BC during the 2011–2018 diagnostic period.

We computed 10-year relative survival rates for each age group for cases diagnosed during the 1990–1999 and during the 2000–2008 periods. Relative survival estimated survival rates associated with mortality from breast cancer by considering time from diagnostic to all-cause death during a 10-year follow-up and by incorporating background mortality, which was obtained from national life tables. Indeed, in population-based studies, the estimation of survival rates using cause of death data can be problematic because the cause of death can be unreliable and treatment-related deaths are not always attributed to the initial disease. Relative survival avoids these problems by considering the total mortality rate as a sum of the expected mortality rate (obtained from national life tables matched on age, sex, and year) and the excess mortality rate associated with BC. Three relative survival models were built for luminal, Her2-amplified, and triple-negative BC. Flexible parametric relative survival models were used, and each model included categorical variables for age at diagnosis (< 40; 40–49; 50–74; ≥ 75) and metastasis at diagnosis [16]. Interaction terms between age groups and metastasis at diagnosis and time-varying effects of age groups and metastasis at diagnosis were also included in the models considering the AIC criterion. We finally computed 5-year relative survival rates by tumor subtypes for cases diagnosed during the 2011–2013 period.

Analyses were realized using Stata 16.1.

## Results

### Incidence

For the period 1990 to 2018, we selected 23,703 cases of invasive BC in the Isere department among whom 1343 were < 40 years at diagnosis.

**Fig. 1:**
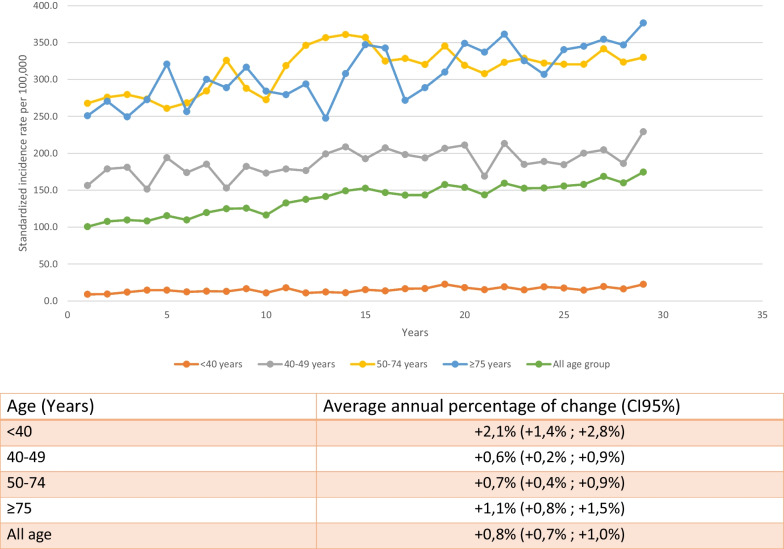
Average annual percentage of change of invasive breast cancer by age at diagnosis from 1990 to 2018 (France, Isere Department)

**Fig. 2:**
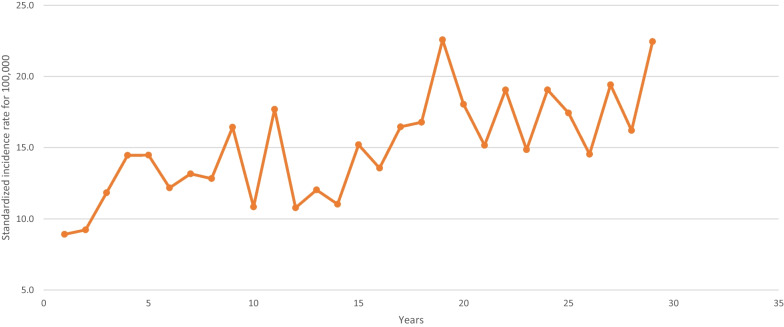
Invasive breast cancer incidence rate for women < 40 years from 1990 to 2018 (France, Isere Department)

Table 1 shows the incidence data for each age group. From 1990 to 2018, the AAPC of invasive BC was + 0,8% per year (95% CI 0,7; 1) for all age groups combined. For the 50–74 years group, targeted by organized screening, AAPC was + 0,7% by year (95% CI 0,4; 0,9) from 267,6 to 329,9 per 100,000. In the < 40 years group, a significant annual increase of 2,1% (95% CI 1,3; 2,8) was observed from 8,9 to 22,4 per 100,000. This is the highest rise in incidence compared to other age groups (Figs. 1,2).

**Table 1:** Standardized incidence rate per 100 000 women year of invasive breast cancer according to age from 1990 to 2018 (France, Isere Department)

	1990	1991	1992	1993	1994	1995	1996	1997	1998	1999	2000	2001	2002	2003	2004
< 40 years	8,9	9,2	11,8	14,5	14,5	12,2	13,2	12,8	16,4	10,8	17,7	10,8	12,0	11,0	15,2
40–49 years	156,3	178,8	181,0	151,5	193,9	173,8	185,2	152,9	182,2	173,1	178,7	176,5	199,1	208,6	192,6
50–74 years	267,6	275,9	279,5	273,4	260,8	268,2	284,4	325,8	287,9	272,5	318,7	346,2	356,6	360,9	356,9
≥ 75 years	250,8	270,4	249,3	272,6	320,8	256,4	300,2	288,9	316,6	284,1	279,4	293,9	247,5	307,9	347,2
All age	100,6	107,6	109,6	108,2	115,4	109,7	119,6	124,8	125,5	116,3	132,6	137,5	141,4	149,1	152,6

	2005	2006	2007	2008	2009	2010	2011	2012	2013	2014	2015	2016	2017	2018
< 40 years	13,6	16,5	16,8	22,6	18,0	15,2	19,1	14,9	19,1	17,4	14,5	19,4	16,2	22,4
40–49 years	207,3	198,2	193,6	206,7	211,0	168,8	213,2	184,9	188,8	184,6	200,1	204,7	186,2	229,2
50–74 years	324,8	328,4	320,3	345,2	319,1	307,8	323,1	328,6	322,1	320,6	320,5	341,4	323,5	329,9
≥ 75 years	342,5	271,7	288,9	310,0	348,9	337,0	361,3	325,3	306,8	340,3	345,0	354,4	346,7	376,5
All age	146,8	143,3	143,4	157,6	153,7	143,6	159,4	152,6	153,0	155,6	157,8	168,6	159,8	174,5

From 2011 to 2018, all age groups combined, there was an increase in incidence of + 0,6% (95% CI − 0,4; 1,6), but the results are not statistically significant. In the < 40 years group, 35–39 year olds accounted for the largest proportion of cases (incidence rate of 81.3 per 100,000 or 60% of cases) and AAPC was + 2,1% (95% CI − 2,1; 6,2). For the 40–49 age group, AAPC was + 1.6% (95% CI − 1.4 + 3.2) with an incidence rate of 163 per 100,000 in the 40–44 age group (Tables 2 and 3).

**Table 2:** Incidence rates by 5-year age group between 2011 and 2018

Age group	Number of case	Population	Incidence rate for 100,000 100 000
0–4	0	303,926	0,0
5–9	0	320,491	0,0
10–14	0	318,004	0,0
15–19	0	310,107	0,0
20–24	5	294,902	1,7
25–29	41	288,357	14,2
30–34	131	312,751	41,9
35–39	265	324,733	81,6
40–44	562	344,732	163,0
45–49	816	348,316	234,3
50–54	883	333,043	265,1
55–59	858	313,374	273,8
60–64	935	297,095	314,7
65–69	1052	263,594	399,1
70–74	864	200,111	431,8
75–79	576	170,320	338,2
80–84	537	150,328	357,2
85 +	598	175,446	340,8

**Table 3:** Average annual percentage of change by age from 2011 to 2018 (CI 95%)

Age group	Average annual percentage of change
< 40 years	+ 2,1% (–2,1%; + 6,2%)
40–49 years	+ 1,2% (–1,4%; + 3,2%)
50–74 years	+ 0,6% (–1,2%; + 1,3%)
≥ 75 years	+ 1,1% (–0,8%; + 3,4%)
All age group	+ 0,6% (–0,4%; + 1,6%)

Regarding tumor subtypes in this period, there were 8.4% triple negatives, 14.3% HER2 amplified, and 74.8% luminal, all age groups combined. Among YW, there were 18.3% triple negative, 25.8% of HER2 amplified, and 54% luminal.

As shown in Table 4, AAPC was -0.8% (95% CI − 4,2; 2,6) for all age groups combined among triple negatives. Nevertheless, there is an increase in the incidence among YW (+ 1,4% by year, 95% CI − 8,2; 11) and those over 75 (+ 4% by year, 95% CI − 5,1; 13,2), but the results are not statistically significant.

**Table 4:** Average annual percentage of change according to age and tumor subtype from 2011 to 2018 (CI 95%)

	Triple negative	HER2 amplified	Luminal
< 40 years	+ 1,4% (–8,2; + 11,0)	+ 6,8% (–1,8; + 15,4)	–0,2% (–5,7; + 5,3)
40–49 years	–4,8% (–12,4; + 2,8)	+ 7,4% (+ 1,1; + 13,7)	–0,1% (–2,8; + 2,5)
50–70 years	–1,3% (–6,1; + 3,4)	+ 3,4% (–0,2; + 7,1)	–0,7% (–2,1; + 0,8)
≥ 75 years	+ 4,0% (–5,1; + 13,2)	+ 12,7% (+ 5,2; + 20,2)	+ 1,1% (–1,3; + 3,5)
All age	–0,8% (–4,2; + 2,6)	+ 6,0% (+ 3,2; + 8,7)	–0,1% (–1,2; + 1,0)

The incidence rate of the luminal group seems to be slightly decreasing for all age groups except those over 75 one.

In the HER2 amplified group, there is a statistically significant increase in the incidence rate of 6% by year in all age groups, 7.4% by year among 40–49-year-olds, and 12.7% among those over 75 years old. Among YW, there is an increase in the incidence rate of 6,8% by year, but the results are not statistically significant.

### Relative survival

**Fig. 3:**
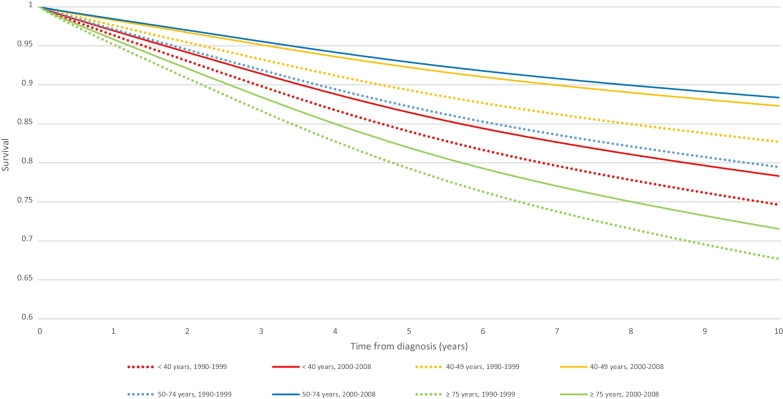
Relative survival for invasive breast cancer according to years at diagnosis by age (France, Isere Department)

From 1990 to 1999, 10-year relative survival was 74,6% (95% CI 69,6; 78,9) in YW group and 79,4% (95% CI 77,7; 81,0) in the 50–74 years group. From 2000 to 2008, 10-year relative survival was 78,3% (95% CI 73,7; 82,1) among women < 40 years and 88,3% (95% CI 87,1; 89,4) in the 50–74 years group. The smallest relative survival is found in the over 75 s at 67% (95% CI 62,2; 72,4) and 71,5% (95% CI 67,1; 73,4) from 1990 to 1999 and from 2000 to 2008, respectively (Table 5). Figure 3 shows the corresponding survival curve.

**Table 5:** 10-year relative survival of breast cancer by age from 1990 to 1999 and 2000 to 2008 (CI 95%)

	1990–1999	2000–2008
< 40 years	74,6% (69,6; 78,9)	78,3% (73,7; 82,1)
40–49 years	82,6% (80,3; 84,7)	87,3% (85,2; 89,0)
50–74 years	79,4% (77,7; 81,0)	88,3% (87,1; 89,4)
≥ 75 years	67,6% (62,2; 72,4)	71,5% (67,1; 75,4)

**Fig. 4:**
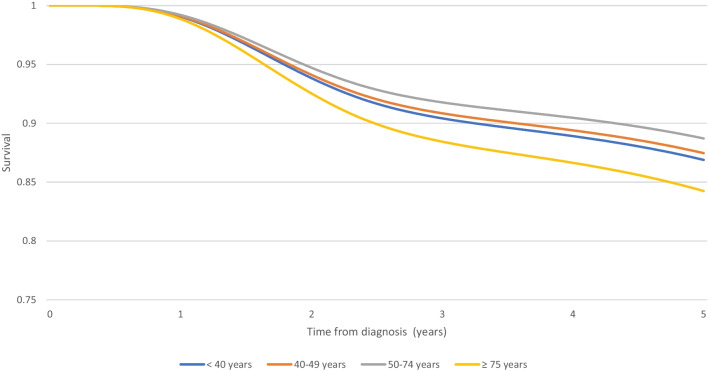
5-year relative survival rates for non-metastatic triple-negative breast cancer among women diagnosed from 2011 to 2013 (France, Isere Department)

**Fig. 5:**
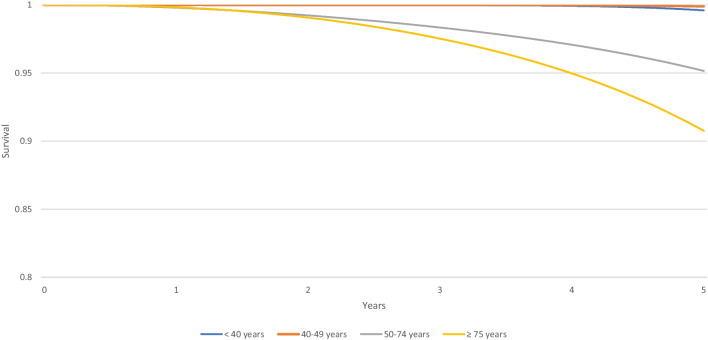
5-year relative survival rates for non-metastatic Her2 amplified breast cancer among women diagnosed from 2011 to 2013 (France, Isere Department)

**Fig. 6:**
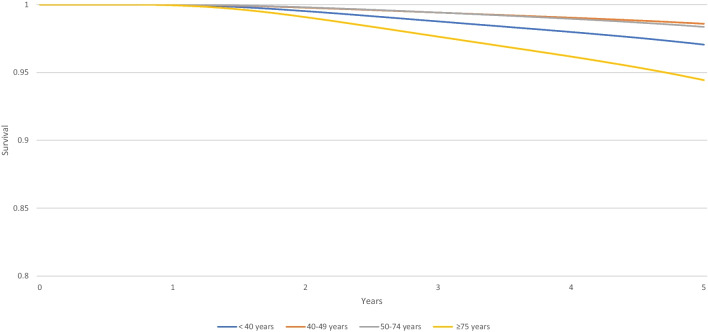
5-year relative survival rates for non-metastatic luminal breast cancer among women diagnosed from 2011 to 2013 (France, Isere Department)

Between 2011 and 2013, 5-year relative survival was slightly lower among triple negative (86,9%, 95% CI 75,6; 93,1) and luminal (97%, 95% CI 94,7; 98,3) in YW compared to other age groups, except those over 75 years old. The 5-year relative survival of YW is higher in HER2 amplified (99,6%, 95% CI 94,7; 99,9) compared to the 50–74-year-old group (Table 6). Figures 4, 5, and 6 show the corresponding survival curve.

**Table 6:** 5-year relative survival of non-metastatic breast cancer by age according to tumor subtype from 2011 to 2013 (CI 95%)

	Triple negative	HER2 amplified	Luminal
< 40 years	86,9% (75,6; 93,1)	99,6% (94,7; 99,9)	97% (94,7; 98,3)
40–49 years	87,4% (79,7; 92,3)	99,9% (96,1; 99,9)	98,6% (97,5; 99,1)
50–74 years	88,7% (83,1; 92,5)	95,1% (90,6; 97,5)	98,4% (97,5; 98,9)
≥ 75 years	84,2% (72; 91,4)	90,7% (79,7; 95,9)	94,4% (91,3; 96,4)

## Discussion

This study provided an update on BC incidence and survival among women in Isere Department from 1990 to 2018. The largest increase in the incidence of BC is found in YW. Our results are consistent with several studies: From French registries, Colonna et al. reported + 0.65% per year of the incidence rate between 1983 and 2002 (15–39 years old) and national data show an increase of 0.9% per year on average from 1990 to 2018 among YW [5, 17]. Leclère et al. have shown an increase in the incidence rate among YW in seven European countries by 1.19% on average per year between 1990 and 2008 [9]. In the USA, Guo et al. have shown a rise in incidence rate among women between ages 20 and 39 years from the Surveillance, Epidemiology, and End Results program’s 9-registry areas from 1975 to 2015, with an annual percent change of 0.5% [18].

We found an increase in the average annual incidence (+ 0,8%) in all age groups over this period. These data are close to those found at the national level in France over the same period, estimated at + 1.1% over the period 1990 to 2018 and + 0.6% between 2010 and 2018 [5]. In the USA, there is a rise in incidence by + 0,3% per year between 2009 and 2018 [19].

This trend in incidence seems to be multifactorial: Management practices can influence BC incidence: In France, BC screening programs have been implemented throughout the country since 2004 but started gradually since other 1994. It is recommended only in women aged 50 to 74 years or if there are risk factors such as a family history or a genetic mutation such as BRCA [6]. The introduction of organized screening could lead to this increased incidence in the 50–74 age group but not totally. Indeed, we also find an increase in incidence in the other age categories. The mechanisms that lead screening to vary incidence rates are the advanced diagnosis effect and overdiagnosis.

A possible explanation for the increase in incidence is the modification in exposure to risk factors of BC: Among modifiable risk factors, several studies have proven a relationship between alcohol consumption and the risk of developing BCYW [20, 21]. Smoking is associated with the risk of developing BC even if this risk is scarce [22, 23]. In France, the increased incidence of smoking in women may partly explain the higher incidence rate of BC [24]. On the other hand, a high BMI is linked to a reduced risk of developing BCYW [25]. Physical activity is also associated with a reduced risk of BC at any age [26].

Regarding hormonal and reproductive factors, the increase in the incidence of BC in western countries could be explained by the decrease in breastfeeding, the later age of the first child with a decrease in the average number of children per woman. Younger age at puberty and later menopause may also explain this trend.

Considerable advances have been made in BC oncogenetic: genetic factors, more frequent in YW must be considered with great importance even if the majority of BCYW are not explained by cancer susceptibility genes [27]. BC predisposition genes BRCA1 and BRAC2 have been known for over twenty years [28, 29]. In the UK POSH cohort, around 12% of women under 41 years old had the BRCA mutation [30]. Therefore, young age alone is sufficient to suggest an oncogenetic consultation. An American cohort study showed that in 2006, 77% of YW with BC had a BRCA test, compared to 95% in 2013, suggesting progress in genetic screening in this category of patients [31]. Later, the mutation of the PALB2 gene was also found to be predisposing to BC [32]. Today in France, the Genetics and Cancer group recommends the analysis of the BRCA 1 and 2, PALB2, TP53, CDH1, and PTEN genes if there is a suspicion of predisposition to BC [33]. Oncogenetic consultation makes it possible to look for predispositions and adapt screening according to these predispositions.

The greatest improvement in survival is found in the 50–74 age group, linked to the implementation of organized screening: Indeed, the diagnosis is made at an earlier stage when treatments are more likely to be effective, but there is also a bias related to the early diagnosis: The date of diagnosis is advanced, and therefore, survival is longer even if the date of death does not change because it is calculated from an earlier date.

We found that YW have lower relative survival than 50–74-year-olds, which is widely described in the literature [34]. However, relative survival is better over time. The latest French national study from the national cancer institute also shows an improvement in survival in all age groups from 1989 to 2018. In YW, the 10-year net survival increases from 72% in 1990 to 85% in 2010 [35]. Because the YW category is not targeted by organized screening, improvement of specific survival is not only linked to earlier screening, particularly in patients with a genetic predisposition, but also to therapeutic advances. Increased awareness of early detection is also an explanation for improved survival. This improvement in survival is found in other European countries [36]. Several explanations can be put forward for the poorer survival among YW: In our study, there are about twice more triple negative among YW compared to the 50–74 age group, which is found in several studies [37]. We know triple-negative BC is associated with a poor prognosis. There were more aggressive tumors compared to older women [38, 39]. There is more HER2 amplified among YW in our study compared to other age groups, but young age has no impact on the relative survival of this type of tumor [40, 41]. Several studies have shown that survival is worse for the luminal subtype in YW [34, 42]. Many mechanisms may explain this finding: Young age is associated with poorer compliance with adjuvant endocrine therapy, and limited efficacy of this treatment [43]. In addition, post-chemotherapy amenorrhea related to hormonal impregnation, associated with better outcomes in this subtype, is less common in YW [44]. Women targeted for screening may have less advanced luminal tumors as a result of screening. Finally, the poorer survival could also be explained by a later stage at diagnosis, but this hypothesis is debated [45, 46]. The lowest relative survival is found in the over 75 s, which may be explained by the higher proportion of comorbidities in this age category and the lower ability to receive standard treatments [47].

BC screening was shown to reduce breast cancer mortality in the randomized controlled trials, and the effectiveness of mammography has been demonstrated in modern screening programs. The International Agency for Research on Cancer estimated that mammography screening reduces breast cancer mortality by about 40% in women ages 50–69 who attend screening [48]. However, several factors could make it difficult to estimate the impact of organized screening since it was introduced, such as increased awareness, the increasing effectiveness of treatments, the modification of risk factors over time implying changes in incidence rates, and the gradual introduction and at a variable level of screening in the different French regions. To overcome the influence of these different factors, the evaluation of screening program data utilizing the incidence-rate-of-fatal breast cancer analysis has demonstrated that as expected, both screening and improvements in therapy have contributed to reductions in breast cancer mortality, but that women who regularly attend screening benefit substantially more from modern treatment advances than women who do not attend screening [49, 50]. The practice of “individual screening” was already widespread in France before the generalization of organized screening even among YW, which could partly explain the increase in incidence in this category. On the other hand, this hypothesis is not sufficient to explain this trend because the increase is greater among YW. Screening for BC has some limitations: In Europe, the risk of being a false positive is 20% for a woman getting tested every two years from 50 to 69 years old, and the risk of having a biopsy when it is a false positive is 3% [51]. However, false positives do not have an impact on the incidence. Nevertheless, overdiagnosis may be partly responsible for the increased incidence, but our study cannot quantify it. According to Hauge et al., the total lifetime risk of radiation BC and the number of radiation-induced BC death were 10 and 1 per 100,000 women, respectively, by having biennial mammographic screening with a latency time of 10 years [52]. However, the use of a biannual mammogram would avoid one death of BC for every 250 women invited [53]. Radio-induced cancers are more frequent among YW than in older women [54]. There are more false positives, and mammography is less efficient for this age category, where MRI or US should be preferred [55].

BCYW is still a rare disease, and screening impact on mortality has not been demonstrated. Thus, BC screening is not advisable for this age class [56]. Nevertheless, by observing the current trend, it seems necessary to ask whether the age of screening should be redefined: Indeed, YW have more aggressive cancers and poorer survival. In our study, the incidence rate in 50–74-year-olds is too high compared to YW: Therefore, it will be difficult to show the effect on mortality of organized screening in younger women. In addition, very high participation will be necessary for screening to be effective. Although the 35–39 age group is similar to the 40–44 age group in terms of disease burden, the incidence rate in this age group is too low to recommend screening. However, studies have investigated the effectiveness of screening between the ages of 40 and 49 with interesting results on mortality reduction: Hellquist et al. demonstrated a statistically significant overall mortality reduction of 29% in women invited to screening compared to those who were not, with an average follow-up of 16 years [57]. In women aged 40–44 years, the mortality reduction was less marked (18%). In their study, Jonsson et al. [58] found a 36% reduction in mortality in the 40–49-year-old group at a median follow-up of 11 years.

In this regard, the latest US recommendations suggest the age of 45 years to start screening since the 45–49 age group is very similar to the 50–54 age group on the absolute risk of breast cancer at 5 years, the proportion of incident cases, and distribution of deaths by age at diagnosis [59].

It could be interesting in the future to consider personalized screening according to the risk of developing BC to screen fewer women at low risk and strengthen the screening of women at higher risk. Indeed, the increase in incidence seen in YW does not result in incidence rates high enough to consider screening in the general population. On a European scale, the MyPeBs study launched in 2019 is evaluating a new BC screening strategy aimed at demonstrating that stratified screening reduces advanced BC and disadvantages of screening (overdiagnosis, false positives). MyPeBs could prepare future screening recommendations in Europe. In France, individualized strategies by screening program for high-risk women were set up in 2014 by the high health authority as part of the 2009–2013 cancer plan. However, no studies have compared multiple screening strategies in different risk groups on mortality reduction.

Our study has several limitations. First, it is a retrospective study with the disadvantages we know. We used data from the Isere register which is not necessarily representative of other French departments. In addition, even if this work allows several hypotheses, it is a descriptive epidemiological study, which makes it difficult to assess the evolution of risk factors and their consequences on our survival and incidence data. We only had tumor subtypes from 2011, which limited the number of cases for a more powerful analysis. Moreover, we did not have tumor grade and Ki67. Thus, we could not divide the luminal category into groups A or B for more accuracy.

The strengths of our work are multiple: The registry information system, based on general population data, allows for the completeness of invasive BC diagnoses. Moreover, the incidence data were calculated over the last three decades, which allows us to have reliable hindsight in our analysis, and a large number of patients included, allowing for significant power. Finally, the data are based on a multicentric population: The registry used includes several medical centers in different cities throughout the department.

## Conclusion

Our results have shown an increase in the incidence of BC over the past thirty years, particularly affecting YW. It will be necessary to strengthen awareness campaigns on known modifiable risk factors and search for other potential risk factors such as environmental exposure. Progress is still needed in improving the identification of high-risk patients to increase the number of early diagnoses. Further research on the evaluation of personalized screening is required. In the meantime, the continuous improvement of survival thanks to new therapies brings important hope in the management of BC.

## Data Availability

Data supporting the findings of this study are available upon request from the corresponding author.

## Abbreviations

BC: Breast cancer
YW: Young women
BCYW: Breast cancer in young women
CI: Confidence interval
AAPC: Average annual percent change
ER: Estrogen receptor
PR: Progesterone receptor
HER2: Human epidermal growth factor receptor type 2
